# Supplementary material for: Higher Body Fat but Similar Phase Angle Values in Patients with the Classical Form of Congenital Adrenal Hyperplasia in Comparison to a Control Group
Source: Nutrients. 2022 Dec 6;14(23):5184. doi: 10.3390/nu14235184 (PMC9739276; doi:10.3390/nu14235184)
Supplement: Supplementary file 1 [file nutrients-14-05184-s001.zip › nutrients-2016622-supplementary.pdf]

Supplementary table S1. Detailed pharmacological therapy of patients with CAH21OHD

Patient	Age	Sex	Phenotype	BMI (kg/m²)	Therapy	Dose (H/m²)	Andro (ng/mL)
1	19	f	sw	28.78	HC + DXM + F	9.45	-
2	19	f	sw	27.18	HC + DXM + F	9.73	1.36
3	20	f	sw	21.84	HC + DXM + F	9.20	1.81
4	29	f	sw	19.41	HC + DXM + F	15.19	1.38
5	27	f	sw	20.55	PRED + F	15.36	1.09
6	20	f	sw	24.09	HC + DXM + F	16.28	1.70
7	22	f	sv	24.15	PRED	12.00	1.17
8	19	f	sv	16.95	HC + DXM + F	12.43	0.43
9	25	f	sv	33.24	HC + DXM	7.60	0.12
10	23	f	sw	22.30	HC + F	23.58	0.12
11	30	f	sv	26.88	HC + DXM	11.08	2.29
12	18	f	sv	29.34	PRED	19.78	5.01
13	23	f	sw	24.93	HC + DXM	8.59	4.74
14	24	f	sv	33.94	HC + DXM + F	8.89	4.94
15	24	f	sv	26.67	PRED	19.28	3.30
16	29	m	sv	32.72	HC + DXM	8.99	0.96
17	28	m	sv	25.89	PRED	15.68	0.66
18	18	m	sv	22.70	HC + DXM + F	10.61	1.98
19	25	m	sw	18.49	HC + DXM + F	12.72	1.54
20	26	m	sv	24.17	HC + DXM	9.71	1.63
21	18	m	sw	25.08	HC + DXM + F	13.22	2.97
22	23	m	sv	29.35	PRED	16.73	8.74

HC, hydrocortisone; DXM, dexamethasone; PRED, prednisone; SW, salt wasting; SV, simple virilizing; Andro, androstenedione; F, fludrocortisone

Supplementary table S2. Characteristics of patients according to vector position on tolerance ellipses based on a healthy reference population

	Patient	Weight (kg)	BMI (kg/m²)	LST (kg)	%FM	LSTI	Xc/H (Ω/m)	R/H (Ω/m)
**Female**								
**Vectors outside 75% to the left of the major axis**	15	60.4	26.67	40.1	29.3	17.7	38.5	300.3
	12	61.1	29.34	34.4	40.3	16.5	53.4	369.4
	11	67.1	26.88	38.4	39.3	15.4	57.0	380.4
	1	68.7	28.78	40.1	38.7	16.8	58.3	392.9
	7	63.0	24.15	35.4	39.7	13.4	58.9	460.7
**Vectors outside 75% to the right of the major axis**	4	48.1	19.41	26.6	41.0	10.7	48.3	436.5
	5	44.1	20.55	29.2	30.7	13.5	51.2	458.7
	3	54.8	21.84	33.5	35.0	13.3	51.8	462.1
	8	44.2	16.95	29.6	30.2	11.3	45.8	468.7
**Vectors within 75% of tolerance**	2	62.8	27.18	33.7	43.3	14.6	46.7	380.3
	6	52.4	24.09	35.3	29.0	16.2	47.5	385.1
	10	50.5	22.30	32.7	31.3	14.4	43.2	370.8
	14	91.5	33.94	42.5	50.1	15.8	42.6	352.6
	13	69.7	24.93	42.1	36.0	15.1	41.3	361.2
	9	73.0	33.24	39.2	43.9	17.8	38.5	326.6
**Male**								
	**Patient**	**Weight (kg)**	**BMI (kg/m²)**	**LST (kg)**	**%FM**	**LSTI**	**Xc/H (Ω/m)**	**R/H (Ω/m)**
**Vectors outside 75% to the left of the minor axis**	20	65.0	24.17	51.4	18.5	19.0	40.1	256.5
	16	74.5	32.72	43.7	37.9	19.2	49.7	298.2
	17	77.5	25.89	50.8	31.0	17.0	50.8	309.5
**Vectors outside 75% to the right of the major axis**	19	44.7	18.49	35.2	16.6	14.6	55.3	421.9
	18	50.4	22.70	29.4	37.7	13.2	55.7	437.6
**Vectors within 75% of tolerance**	21	69.5	25.08	42.2	36.0	15.2	42.6	333.9
	22	75.6	29.35	53.0	27.9	20.6	34.9	251.1

LST, lean soft tissue; FM, fat mass; LSTI, lean soft tissue index; Xc/H, reactance/height; R/H, resistance/height.
